# Tumour Evolution and Seed and Soil Mechanism in Pancreatic Metastases of Renal Cell Carcinoma

**DOI:** 10.3390/cancers13061342

**Published:** 2021-03-16

**Authors:** Franz Sellner, Sabine Thalhammer, Martin Klimpfinger

**Affiliations:** 1 Department of Surgery, Medical University of Vienna, 1090 Vienna, Austria; 2 Department of General-, Visceral- and Vascular Surgery, Clinic Favoriten—Kaiser Franz Josef Hospital, 1100 Vienna, Austria; sabine.thalhammer@gesundheitsverbund.at; 3 Clinical Institute of Pathology, Medical University, 1090 Vienna, Austria; martin.klimpfinger@meduniwien.ac.at

**Keywords:** renal cell carcinoma metastases, isolated pancreatic metastases, risk factors, tumour evolution, seed and soil mechanism

## Abstract

**Simple Summary:**

The occurrence of pancreatic metastases, especially isolated pancreatic metastases (PM) in metastatic renal cell carcinoma, is associated with unusual clinical features—such as favourable prognosis and ineffectiveness of tumour volume and growth rate dependent risk factors. On the other hand, it was hypothesised that the entity of isolated pancreatic metastases should be considered as a pattern of “seed and soil” mechanism. An extensive literature search was conducted to investigate whether and what mechanism links these two observations. The result of the study shows that an unusually strong seed and soil mechanism (SSM), which allows metastases settlement only in the pancreas, inevitably leads to the ineffectiveness of these risk factors in isolated PM. The hypothesis that the entity of isolated PM in renal cell carcinoma (RCC) is to be regarded as a paradigm for an SSM is thus further supported, and the interaction with the two-phased cancer evolutionary model is discussed.

**Abstract:**

In metastatic renal cell carcinoma, pancreatic metastases can appear in two clinical manifestations: (a) very rarely as isolated pancreatic metastases and (b) in the context with multi-organ metastatic disease. Both courses are characterised by rare, unusual clinical features. For isolated pancreatic metastases, the literature shows no effect on survival in all 11 publications that examined the effect of singular versus multiple pancreatic metastases; a lack of effect on survival time was also present in all 8 studies on pancreatic metastases size, in 7 of 8 studies on the influence of disease-free interval (DFI), and in 6 of 7 studies on the influence of synchronous versus metachronous metastases. In multi-organ site metastases observations, on the other hand, all five available references showed significantly better results in patients with concurrent pancreatic metastases compared to those without pancreatic metastases, although the total number of affected organs in the pancreatic metastases cohort was larger. Tumour volume-dependent risk factors thus remain surprisingly ineffective in both groups, which contradicts the usual behaviour of solid tumours. The reasons for this unusual behaviour and possible relations to tumour evolution and the hypothesis of an influence of a seed and soil mechanism in the occurrence of pancreatic metastases in metastatic renal cell carcinoma are discussed.

## 1. Introduction

The continuous evolution of life is triggered by two mechanisms: on the one hand by genetic alterations occurring from generation to generation and on the other hand by the proving or non-proving of the resulting altered biological properties of the organisms in confrontation with their respective environment. Genetics thus provides for larger or smaller variations in living beings and the environment for the selection of the best adapted organisms. Malignant tumours—i.e., newly emerged cell clones that are genetically in some aspects different from the organism of origin and independent of its control mechanisms—can also be regarded as “new organisms” within an organism in a somewhat exaggerated way [1,2,3,4,5,6]. It is therefore not surprising that both mechanisms also play an important role in the metastasis behaviour of carcinomas. The genetically determined development of tumour cells capable of metastases is currently explained by two models. The traditional linear stepwise “Neo-Darwinian” clonal expansions model [6], which has been accepted for a long time, has recently been contrasted with a two-phase cancer evolutionary model [5,6,7,8] consisting of genome reorganisation [5] mediated punctuated macro-evolution, followed by gene mutation/epigenetic-based stepwise micro-evolution [4,6].

At the beginning of metastasis development, there is an intravasation of genetically different cells with different biological properties from the primary tumour into the vascular system. Subsequently, a multistep cascade process requires numerous matching interactions of embolised tumour cells and host organs for successful tumour cell colonisation. Thus, the clinical properties of metastases are the common end product of tumour and host biology. Since the properties of the circulating tumour cells vary according to the primary tumour, as do the properties of the various host organs, this results in a selection of suitable tumour cells for metastasis settlement and in a non-random distribution of the metastases in various host organs.

## 2. Seed and Soil Mechanism

Paget [9] first recognised the non-random distribution of metastases in 1889 and described the phenomenon as “seed and soil mechanism” (SSM), which aptly explains the fate of embolised tumour cells by an interaction of properties of tumour cells (seed) with corresponding properties of host organs (soil). The two-phase cancer evolutionary model mentioned at the beginning, which combines the phase of a genome mediated, punctuated cellular macro-evolution and a subsequent phase of a gene mediated stepwise micro-evolution, can of course also serve to describe and explain the relationship between “seed and soil”. First, the punctuated macro-evolution phase leads (by chromosomal chaos—chromosomal re-organisation [4]) to the generation of new genomes and thus “new” cell species of great heterogeneity [6] with different metastatic properties (seed). Subsequently, the stepwise micro-evolution phase allows the growth of suitable tumour cell populations to metastases only in interaction with suitable host organ/tissue (soil). Thus the “new” seed can be understood as a newly formed genome by macro-evolution while the “suitable” soil is the result of environmentally defined micro-evolutionary selection: a seed dominated phase is followed by a soil dominated phase.

Since neither each embolised tumour cell nor each host organ can provide all the necessary biological properties required for successful interaction, this leads to a tumour cell selection in the early onset of metastatic colonisation with a subsequent organotropism. Studies in recent decades have elucidated this early metastatic process and demonstrated it as a multi-stage, cascade-like evolutionary process (pre-metastatic niche formation, intravasation, transport, docking, extravasation, and colonisation) that takes place through a variety of successful interactions of tumour cell and host organ properties [10,11,12,13,14,15,16]. In human medicine, the efficacy of the SSM was for a long time limited to the realisation of a distribution pattern of the preferred metastatic organs specific to the different primary tumours. Only in recent years have clinical observations been added, e.g., the absence of countless lung metastases after peritoneo-venous shunt in malignant ascites treatment [17] or the influence of immunosuppressive therapies on the course of tumour diseases [18]. It was therefore surprising when in 2018, with isolated pancreatic metastases of renal cell carcinoma (isPMRCC) known since 1952 [19], an entity was identified [20] that can be addressed as a paradigm for the exquisite effect of an SSM in human medicine. The main argument for the impact of the hypothesis of SSM in the isPMRCC was realisation that there is evidence that the isolated occurrence of pancreatic metastases (PM) takes place in the course of a systemic haematogenic metastasis process and is not due to a direct, local metastatic pathway between the kidney and the pancreas, as previously assumed. However, studies in recent years have also shown that the occurrence of PM in metastatic renal cell carcinoma (mRCC) may be associated with two further, equal unexpected clinical properties: (a) the lack of influence of conventional risk factors for overall survival (OS) in isPMRCC [21] and (b) the better prognosis of multi-organ site metastasised RCC with concomitant PM compared to those without PM [22]. The aim of the presented review is therefore (1) to investigate on the basis of the currently available literature whether these two observed clinical peculiarities are separate independent peculiarities of PM in mRCC, or whether there is a connection with this suspected exquisite SSM and (2) to evaluate the role of the interplay between tumour cell evolution and host organ environment in the clinical course of this entity. To this end, the available literature on these two clinical features is presented and examined.

## 3. Pancreatic Metastases in Renal Cell Cancer

Metastatic cancer to the pancreas from other primary sites is rare. In autopsy studies, the frequency is estimated at 1.6–11% [23,24,25,26]. Of 16,614 pancreatic operations due to malignancy listed in 16 clinical reports [27,28,29,30,31,32,33,34,35,36,37,38,39,40,41,42], only 258 were performed for PM, resulting in a frequency of just 1.6%. Renal cell carcinoma (RCC), lung carcinomas, colorectal malignancies, melanomas, and sarcomas were reported as the most common primary malignancies [23,24,29,43,44,45,46]. With a proportion of over 60%, RCC is by far the most common primary tumour leading to PM [23,29,36,38,43,44,46,47,48,49], with PM being more frequent in the clear-cell subtype [50]. In the course of mRCC, PM may appear: (a) as the sole metastatic site: isPMRCC and (b) in the context with multi-organ metastatic disease.

## 4. IsPMRCC

### 4.1. Clinical Presentation

IsPMRCC are rare observations in which singular or multiple, synchronous or metachronous to the RCC distant metastases occur definitely or over a longer period exclusively in the pancreas. Since the last literature overview (*n* = 1034) covering the years 1952 to 2019 [51] a further 173 isPMRCC observations could be added [27,28,52,53,54,55,56,57,58,59,60,61,62]. These 1207 observations serve as a basis for a literature review with meta-analysis.

In addition to the sole occurrence of PM, the clinical course is further characterised by: (1) a long interval between primary RCC therapy and the occurrence of PM. An interval of 9.6 (SD 6.8) years was calculated from 470 individually documented observations [28,51,52,53,55]. In single institution reports [27,30,32,33,34,39,40,41,44,47,57,58,59,60,61,62,63,64,65,66,67,68,69,70,71,72,73,74,75,76,77,78,79,80], values from 5.3 [34] to 14 years [27] are documented (Table S1); the longest interval reported so far is 36 years [81]; (2) the frequent occurrence of multiple PM: Of 592 documented cases, 39.2% were multiple PM, with a mean of 3.1 pancreatic foci [82] and a maximum of 15 foci [28], and in large case compilations [27,30,33,34,35,39,41,47,56,57,63,68,71,72,75,76,77,78,79,83,84], values of 9% [71] to 70% [39] are given with a median of 36% (Table S2). (3) by favourable treatment results for metastasis surgery. From 445 isPMRCC cases, a cumulative 5-year survival rate of 74.2% was calculated. In single institution reports [24,27,28,30,31,32,34,35,39,41,42,43,44,45,46,47,56,57,64,66,67,68,69,71,74,75,76,78,79,83,84,85], values from 43% [78] to 100% [85] are presented with a median of 71%, and for the median overall survival following PM treatment, (OS) values from 3.4 [74] to 8.75 years [34], with a median of 6.2 years (Table S3) are reported. Further metastases after isPMRCC surgery [68] were observed in 124 [21,23,28,33,34,39,42,52,53,55,70,71,73,76,79,80,84,86,87,88,89,90,91,92,93,94,95,96,97,98,99,100,101,102,103,104,105,106,107,108,109,110,111,112,113,114,115,116] (Table S4) out of 288 adequately documented observations (43%) after a median interval of 29.8 months (maximum 132 months [56]).

### 4.2. Local or Systemic Metastatic Pathway

Since these data have already been published [20,51,82] they are presented here in an abbreviated form. The exclusive occurrence of metastasis in the pancreas is difficult to reconcile with a systemic haematogenous tumour cell seeding at first glance. It is very unlikely that per pure chance all embolised tumour cells are transported exclusively to the pancreas when considering the small amount of blood flowing through the 120–180 g of pancreatic tissue. This is even more true for multiple tumour cell embolies, which must have preceded the multiple PM observed at 39%. Therefore, for years, authors who reported on this entity put up direct local metastatic pathway (MP) for discussion. This is on the one hand a local-lymphogenic MP after regional tumorous lymph node (LN) blockade [32,42,80,83,86,117,118] and on the other hand, a local venous MP via innate reno-portal anastomoses [119] or via acquired draining collateral veins of hyper-vascularised tumours [32,42,80,83,86,87,117,118,120,121]. However, a high importance of these two MPs is countered by the fact that there are no reports of more frequent peripancreatic LN metastases in isPMRCC—that would be expected in lymphogenic tumour cell propagation—nor are there more frequent postoperative liver metastases that would be expected to be associated with a high impact of reno-portal anastomosis in tumour cell spread. For the frequency of peripancreatic LN metastases (Table S5), a value of 6.2% is calculated from 481 observations [28,30,32,35,41,43,56,57,68,69,71,73,75,76,84,88,122,123], and for the frequency of later liver metastases (Table S6) a value of 8.7% can be obtained from 288 observations documenting the postoperative outcome [21,28,33,34,42,70,80,84,87,89,90,91,92,93,94,124]. These results attribute little if any importance to a local MP, whether lymphogenic or venous.

To clarify the question of a greater significance of a systemic or a local MP, epidemiological investigations are suitable [51]. Since the local MP should offer for right-sided RCC a greater chance of metastasis in the near caput pancreatis, while for left-sided RCC the chance of metastasizing into the nearby corpus and cauda region is greater, a dependence of metastasis localisation in the pancreas from the RCC side should result. Conversely, diffuse cell spread in a systemic haematogenous pathway should lead to a distribution pattern of PM independent of the RCC side. The epidemiological data demonstrate a uniform distribution of metastases in the pancreas [51] and in particular a significant independence of the distribution from the side of the primary RCC (Table 1). This result has now also been confirmed in large single and multicentre studies [30,35,38,43,69].

The impact of the systemic MP is further underlined by the fact that of the few metastases (*n* = 45) that were diagnosed and successfully operated before the occurrence of PM (Table S7), 78% were undoubtedly of systemic haematogenous origin [23,28,34,36,39,70,71,75,84,86,93,95,96,97,98,118,125,126,127,128,129,130,131,132,133], as were 75% of the 124 metastases that occurred after removal of PM [21,23,28,33,34,39,42,52,53,55,70,71,73,75,79,80,84,86,87,88,89,90,91,92,93,94,95,96,97,98,99,100,101,102,103,104,105,106,107,108,109,110,111,112,113,114,115,116] (Table S4). Thus, the epidemiological data available so far from isPMRCC give only minor importance to a locally lymphogenic or venous metastatic pathway but can be reconciled with a high impact of a systemic haematogenic MP.

## 5. IsPMRCC and the Impact of Prognostic Factors on OS

In older literature there are reports that with synchronous and especially multiple isPMRCC a surgical therapy is not meaningful, since these—analogous to the experience with other solid tumours—were regarded as a negative prognosis criterion with a poor OS [99,100,117,125,134]. In a literature compilation in 2006 [21], our working group demonstrated, however, that the treatment results are not influenced by the singular or multiple or by the synchronous or metachronous occurrence of the PM. The current literature search revealed 12 large (*n* > 20) institutional reports and compilations [30,35,38,47,51,56,57,65,68,69,72,111] in which data on the impact of clinical risk factors on the OS of operated isPMRCC were presented (Table 2). (The institutional reports were all retrospective and spanned multi-year periods (15–37 years). All studies were performed in surgical departments and inclusion criteria were uniformly surgically treated isPMRCC. Information on the histological subtype was given in four papers: a total of 144 clear cell tumours were compared with only three papillary and one chromophobe. Detailed gene expression analysis and mutation profiles have not yet been presented in isPMRCC).

The influence of single vs. multiple PM on the OS was calculated in 11 studies, all of which yielded a negative result. The influence of tumour size was documented in eight publications, all of which were also negative; the disease-free interval (DFI) between RCC and PM surgery was analysed eight times, with one exception always with a negative result. Finally, the influence of synchronous vs. metachronous PM was investigated in seven papers, six of which yielded a negative result. Of a total of 34 analyses investigating the influence of clinical risk factors such as multiple PM, size, synchronous PM, and DFI, 94.1% thus produced a negative result. The analysis of peripancreatic lymph node involvement revealed a contrary result. Investigated in seven publications, an impact on the OS was identified four times. These LN metastases apparently indicate the beginning of the transition from isPMRCC to multiple organ site metastases with poor prognosis in a relevant proportion of observations. Finally, a possible influence of staging and grading of the primary tumour has been investigated so far only by Di Franco [56], who reported a lack of influence. A study of the influence of the IMDC score [135] or molecular markers in isPMRCC was not found in the cited works.

In summary, the literature review confirms that clinical prognostic factors that reflect tumour burden and tumour growth rate, such as size, occurrence of multiple metastases, DFI, or synchronous occurrence of PM, which are generally relevant in metastasis surgery of solid carcinomas [136,137,138], remain ineffective with isPMRCC. In particular, the complete lack of influence of the tumour volume on survival contradicts a general concept of cancer, in which a greater tumour burden of the body results in a poorer outcome [139].

## 6. Pancreatic Metastases and OS in Multi-Organ Metastatic RCC

In early studies, PM in multi-organ metastases of RCC were often classified as terminal events, with poor forecast due to the lack of an effective drug therapy [101]. Only advances in the drug therapy of the RCC—the introduction in clinical praxis of targeted therapies with antiangiogenetic agents and immunotherapy [140,141,142]—have increasingly sparked interest in the actual frequency, the course, and the clinical relevance of these PM and initiated further investigations. The results (Table 3) unexpectedly show that patients with multi-organ site mRCC and concomitant PM have more favourable survival times than those without PM [22,58,67,139,142]. (The design of the five studies was retrospective four times and was unspecified once. The inclusion criteria were three times a first line anti-angiogenetic therapy, once all PM diagnosed in the observation period, and once were not specified. Detailed genetic and molecular markers analyses have only been reported once [58]).

In 2013, Grassi [22] reported for the first time that in mRCC the median OS was more favourable for patients with PM at 39 months than for those without PM at 23 months (*p* = 0.0004), an observation made by Yuasa [67] in 2015 reaffirmed. Kalra [142] confirmed this result in a comprehensive study again in 2016: a median OS of 39 months for patients with PM corresponded to 26 months for patients without PM (*p* < 0.01), although the total number of affected organs was greater in the collective with PM (*p* < 0.001). Chrom [139] also observed a better OS for the group of mRCC with pancreatic involvement: 46 months vs. 23 months, which was significant in univariate analysis (*p* = 0.022) but did not prove to be an independent variable in multivariate analysis. In a recent study, Singla [58] compared mRCC with and without presence of PM with regard to clinical behaviour and underlying biology. In doing so it was reaffirmed that the cohort with PM had a more favourable OS than the one without PM (OS 101 vs. 35 months (*p* < 0.001), 5-year survival rates 88% vs. 31%, *p* < 0.001). There was also a high sensitivity of the cohort with PM to antiangiogenetic agents with a disease-free survival of 26.9 vs. 8.3 months (*p* = 0.007) and a simultaneous resistance to immune checkpoint inhibitors with more rapid progress of PM observations (*p* = 0.034). Finally, within the PM cohort, Singla noted an independence (*p* = 0.684) of survival from the established IMDC score, which is not based on pathological–anatomical parameters. This observation, however, is in contrast to Yuasa [67], who observed in the PM group a significant difference in OS between good and intermediate IMDC risk patients (*p* = 0. 013). As a summary, all five investigations carried out so far consistently show that the presence of PM exerts a positive influence on the OS in mRCC.

However, in the three publications [22,139,142] (Table 3) that reported on the possible influence of the number of organs affected by metastases, it was consistently shown that the proportion of single organ metastases was smaller in mRCC with PM than in the respective non-PM control group, and vice versa, the rate of observations with three or more affected organs in the PM group was higher than in the control group. The cohorts with PM were thus consistently characterised by a reduced proportion of prognostically favourable single organ metastases and an increased proportion of prognostically unfavourable multi-organ metastases. In the study of Kalra [142], the difference even reached significance level (*p* < 0.001). Thus, the paradox is that in the mRCC, the presence of PM is associated with more favourable OS, although the total number of affected metastatic organ sites is greater. Therefore, an unconscious selection bias of favourable cases with lower number of metastatic organ sites in the PM group has to be excluded as the cause of the more favourable prognosis. The better prognosis, despite the higher rate of affected organs in the PM group, indicates that the occurrence of PM in the mRCC is associated with tumour cell properties, which trigger a more favourable course [22,58,142].

## 7. Systemic Treatment

Over the last two decades, systemic treatment of metastatic RCC has evolved and improved dramatically. With molecular targeted therapies such as multi tyrosine kinase (MTK) and mTor inhibitors and immune checkpoint inhibitors (ICI), an effective therapy of mRCC is available with lengthened progression-free survival and OS [140,141,142,143,144,145,146,147,148,149]. These therapies are used alone or in combination, whereby the treatment recommendations are oriented to the risk profile and are increasingly individualised [149,150]. Due to their rarity, only a few studies are available for the treatment of PM of the RCC and in particular isPMRCC [22,58,139,142,143,151]. These show a good response to MTK inhibitors (median OS ranging from 27 [58] to 56 [151] months), which was even a match for surgical treatment in one retrospective study [143]. This is contrasted with resistance to ICI (median OS 3 months [58]). Both results could be explained by the enrichment of angiogenic markers and low levels of inflammatory biomarkers in PM observations (see Section 9.1 [58]).

## 8. Discussion

IsPMRCC are an exquisitely rare presentation of mRCC. Until 2000, only 97 cases were documented [152], thus only about two cases/year. Only the improvements in pancreatic diagnostics due to dramatic advances in imaging techniques and the impact of follow-up programs have significantly changed this. Until the end of 2019, 1034 observations were presented [51], corresponding to about 45 new observations per year for the last 20 years, and in the last year more than 150 additionally cases have been added.

The impressive total number of observations now available allows for meaningful investigations. An increasing number of institutional and casuistic reports supplies evidence for a diffuse distribution pattern of isPMRCC within the pancreas and especially its independence of the site of the kidney affected by the tumour. This observation contradicts a high importance of a local metastatic pathway but highlights the strong impact of a systemic haematogenic MP in this entity. However, if, despite systemically haematogenic tumour cell spread, the settlement of metastases is successful only in one organ, the pancreas, then this is undoubtedly a non-random distribution. According to our best knowledge, a non-random distribution pattern of metastasis signals the effect of an SSM due to tumour cell selection resulting from interactions between tumour cells and host organs. This hypothesis of the impact of an SSM in isPMRCC was made for the first time in 2018 [20] on the basis of epidemiological observations (*n* = 666), which could be confirmed here in a larger group (*n* = 1207). This hypothetical SSM is triggered by an interplay of host organ characteristics with specific tumour cell properties acquired during the course of tumour evolution. This SSM manifests itself in two interrelated peculiarities: (1) it allows the embolised tumour cells only in the pancreas to successfully settle and mature into clinically manifest metastases, and (2) in all other organs, however, the settlement and growth of tumour cells to metastases is either made impossible at all, or a dormant state of embolised tumour cells is forced for years [15,20,153]. This high selectivity and effectiveness are unusual because, in general, a SSM causes a relative preference or rejection of individual host organs in metastasis formation. In the case of isPMRCC, however, there is an (almost) absolute effectiveness of host organ preference or rejection, which leads to the exclusive occurrence of metastases in the pancreas.

The influence of tumour volume and growth rate dependent risk factors on the OS of isPMRCC was presented in 12 high volume (*n* ≥ 20) publications. A total of 34 detailed analyses showed the impressive result that in 94% these risk factors remain ineffective in isPMRCC. This uniformity of results is a clear indication that the ineffectiveness of these risk factors cannot be attributed to chance due to the still small number of cases but is another characteristic of isPMRCC. This unexpected lack of prognostic significance of tumour volume and growth rate-dependent risk factors in isPMRCC cannot only be explained by the hypothesis of a strongly effective SSM, but this SSM virtually enforces this behaviour: in principle these risk factors are only an expression of the likelihood that after PM resection of occult micrometastases in other organs, a generalisation stage will take place later [51]. However, since the absolute efficacy of SSM in isPMRCC implies that no metastatic tumour cells outside the pancreas can settle and mature into metastases, these risk factors must inevitably remain ineffective. Therefore, the ineffectiveness of the suspected risk factors does not constitute an accidental second peculiarity of isPMRCC but is an inevitable consequence of the equally selective as effective SSM and represents a further argument for the hypothesis of the impact of an SSM in isPMRCC.

Of course, the absence (respectively the inhibited growth over years) of occult, extrapancreatic tumour cell nests also co-explains the favourable treatment results of the isPMRCC with a 71% 5-year OS rate. These favourable results are matched by the fact that no further metastases were observed later on in 57% of the patients, while the remaining 43% showed subsequent tumour progression, 15.3% of which (Table S4) appeared as further isPMRCC in the pancreas remnant [21,33,52,55,71,76,80,84,87,98,113,114,115]. This suggests that in these few cases, tumour cells have a long-term biological stability [58] that favours the reoccurrence of metastases in the pancreas. In the majority, however, the occurrence of metastases anew signals the evolution to more aggressive cell clones, which trigger conventional tumour generalisation with multiple organ site metastases.

The unanimously in five publications confirmed result of better treatment outcomes for patients with multi-organ site mRCC and concomitant PM compared to those without PM must also be regarded as surprising. This phenomenon cannot be due to an unconscious selection of more oligometastatic cases in the PM group, since in the publications reported so far, the number of affected organs in the PM collective was even greater [22,139,142]. Recent genetic studies (see Section 9) rather suggest that the more favourable OS in mRCC with PM is due to the occurrence of tumour cells with lower aggressiveness [58,154] that may rarely arise during RCC evolution. However, the clinical observation of the increased total number of affected organs in the PM cohort is contradictory, since this is difficult to reconcile with a reduced aggressiveness. The clinical course thus provides two contradictory findings: an increased susceptibility of the organs to metastases, which can be attributed to an increased aggressiveness, and a slower growth with more favourable OS, which indicates a reduced aggressiveness. Two mechanisms can be used as a possible explanation: (1) In the triggering RCC clones, the ability to metastasise is completely separate from the ability to grow more rapidly, so they act as two independent properties, and (2) The tumour cells are characterised by a lower aggressiveness; however, the tumour cell settlement is modified by the same SSM postulated in the isPMRCC. Only the strength of this SSM may be differently pronounced. In the course of multi-organ metastatic RCC disease with concomitant PM, this reduced growth capacity of extrapancreatic metastases is weakly pronounced and causes only an impairment of the growth of extrapancreatic metastases and thus a better OS for the observations with multiple-organ metastases and concomitant PM compared to those without PM. In the case of isPMRCC, the restriction of extrapancreatic colonisation and growth is so pronounced that embolised tumour cells cannot settle and mature to manifest metastases extrapancreatically, which leads to the isolated occurrence of PM and the very favourable OS.

### Limitations

Besides the long observational period with important advancements of surgical and medical therapies and the overall small individual sub-collectives, limitations include the retrospective nature of the analysed papers and the possibility of a selection bias.

## 9. Pathomechanisms Leading to isPMRCC

### 9.1. Genetics and Micro-RNA

After the genome of clear cell RCC (ccRCC) was elucidated in 2013 [155], studies addressing biochemical alterations affecting the metastatic behaviour of ccRCC appeared just a few years later [58,154,156]. In a comprehensive study by Turajlic [154], three factors were presented as important for the metastatic potential of RCC: (a) Two chromosomal alterations were found to be enriched in ccRCC metastases clones: 9p21.3 loss and less pronounced a loss of 14q31.1, which are highly specific events driving metastasis; (b) Low intratumor heterogeneity and low amount of tumour genome affected by somatic copynumber alterations diminish metastatic potential of primary RCC; (c) Distinct patterns of metastases are caused by punctuated and branched evolution. More interesting for the analysis presented here, however, are three cases of isPMRCC presented in detail in the results. First, these observations also confirm the well-known clinical phenomenon of long latency between RCC treatment and occurrence of PM. However, genetic analyses revealed a fundamentally different behaviour of isPMRCC observations compared to extrapancreatic RCC metastases: isPMRCC were associated with lack of 9p loss and a significant lower weight genome instability index (*p* = 0.0489). In the same year, Singla [58] presented comprehensive genetic analyses in PM of the RCC and proved alterations in the PM group associated with a lower aggressiveness (low frequency of BAP1 mutations, which is associated with aggressive disease and a high frequency of PBRM1 loss (>75%), which is associated with less aggressive disease [149,157,158,159] and with a low frequency of copy number alterations associated with aggressiveness, such as 9p, 14q, and 4q loss). The occurrence of isPMRCC is thus linked to cell clones with lower aggressiveness, which differ from clones responsible for conventional generalisation stages of RCC by lack of loss of 9p, lower weight genome instability index, low frequency of BAP1 alterations, and a high frequency of PBRM1 loss. Finally, the authors reported that the PM and the corresponding primary tumours were enriched for angiogenic markers and had low levels of inflammatory markers (based on IMmotion and COMPARZ gene signatures), likely explaining sensitivity to antiangiogenic therapies and insensitivity to ICI [58].

The reduced aggressiveness of these cell clones also explains the good prognosis of isPMRCC. However, the low aggressiveness alone cannot explain the exclusive occurrence of metastases in the pancreas in the absence of metastases in all other organs. This selective behaviour rather suggests an additional influence of a selection mechanism: an SSM, the occurrence of which in isPMRCC finds support in the epidemiological data presented. The inevitable necessary trigger for the formation of isPMRCC would of course be the evolution of tumour cells into clones with special and unusual characteristics: on the one hand these cells are endowed with a reduced aggressiveness determined by proven genetic alterations. On the other hand, in the course of tumour evolution these cells have also taken on properties that trigger in different host organ a highly specific SSM, which allows metastasis settlement only in the pancreas.

Due to their exquisite rarity, no studies regarding the underlying pathomechanisms for SSM in isPMRCC have been presented so far. Therefore, at present, only analogical conclusions of more frequent and better studied large tumour entities can serve as a substitute.

The effect of an SSM brings about that those tumours will gain an advantage in metastasis settlement that produce a variety of cells with different metastatic potentials, as this increases the likelihood that a suitable tumour cell reaches a suitable host organ [20]. RCC is characterised by a high heterogeneity [140,155,160], for which a multitude of microRNA is co-responsible [161,162,163,164,165,166,167,168,169,170,171,172,173,174]. MicroRNAs control the metastasis capability through their ability to inhibit target genes involved in early steps of the metastatic cascade, e.g., epithelial–mesenchymal transition, migration, and metastasis settlement. The numerous interactions of these microRNA in individual tumour cells result in manifold capabilities for metastasis which increases the probability that one of the embolised tumour cells exactly “fits” the properties of the target organ [51]. The impact of microRNA is further emphasised by different microRNA profiles in metastatic and non-metastatic RCC [175,176] as well as by the dependence of microRNA profiles from the location of distant metastases in lungs, bones, or brain [177].

### 9.2. Mechanism Leading to Organotropism

In principle, non-random distribution among distant organs, i.e., organotropism, will occur during metastases colonisation if the metastasis process requires successful interactions between properties of the tumour cells and those of the host organ [178]. Several such mechanisms are already known, such as the pre-metastatic niche formation (pmN), organ-specific differences in immunosurveillance, the chemokine (CK) receptor mechanism, and metabolic adaptation of tumour cells (Table 4).

The term pmN describes the influence of a primary tumour on potential host organs that occurs prior to the arrival of circulating tumour cells to create a “fertile soil” that supports seeding, colonisation, survival, and outgrowth of metastatic tumour cells [14,179,180,181]. The pmN is prepared by the interaction of primary tumour-derived components [11,178,182], tumour-mobilised bone marrow-derived cells (BMDC) [178] and local host organ microenvironment [180,181]. These components control the formation of pmN in a stepwise evolutionary process. In the first initiating step, tumour-released soluble factors and microvesicles [178,183] trigger the activation and recruitment of BMDC (e.g., myeloid-derived suppressor cells, tumour-associated macrophages, regulatory T cells) into the later pmN [178,181]. In a second step, these alter the biological properties of the host tissue environment in such a way that, thirdly, a microenvironment is established in the pmN that creates a fertile soil for incoming tumour cells through inflammation, immunosuppression, increased angiogenesis, vascular leakiness, and extra-cellular matrix remodeling [178,184]. The evolution of pmN thus occurs in a well-defined sequence of events, which are controlled by the interaction of tumour cell and host organ properties. Since the profile of these properties varies in different primary tumours as well as in different host organs, this inevitably triggers an organotropism that is a characteristic of pmN [181]. For RCC, the formation of a pmN—albeit, in the lungs—was already documented in 2011 [185]; to the best of our knowledge, the formation of a pmN of the RCC in the pancreas has not been documented so far.

The impact of immune defense in RCC was first recognised by cases of spontaneous metastasis regression in RCC, at that time interpreted as the effect of enhanced immune defense [96,186]. The recent treatments with immune checkpoint inhibitors [114,141,187,188] such as anti-PD-1 or anti-CTLA-4 highlights the importance of immune competent cells in RCC metastasis settlement. Immune suppressive cells help cancer cells to evade immunosurveillance and to improve tumour cell survival in circulation but also to prepare the formation of pmN in potential host organs [11,181]. Different organs, for example, harbour unique tissue-specific resident immune cells that are involved in metastasis [189,190,191,192,193]. The local heterogeneity of tumour immune landscapes can also be attributed to the tumour epithelial–mesenchymal plasticity in modulating antitumour immunity [183,184]. This difference in equipment and function [188,189,190,191,192,193] will result in organotropism in metastasis settlement, too, because individual organs may impose different selective pressure on metastatic cancer cells [194]. In this context, it is interesting to observe that the immunotherapy generally effective in mRCC is ineffective in isPMRCC [58], which indicates a different immunological behaviour of isPMRCC.

A successful interaction between a CK receptor on the tumour cell surface and a suitable ligand on the host organ is a prerequisite for the activation of numerous signal transducing pathways which are critical in the early metastatic process [195]. Since the CK receptor is tumour cell-specific and the appropriate ligand host organ-specific, this mechanism leads to organotropy, e.g., the CXCR4 receptor expressed on human breast cancer cells has been demonstrated to facilitate the metastasis of these cells to the lung, an organ rich in the corresponding ligand CXCL12 [13,196].

Organotropism can also be caused by the necessity for the metastasizing tumour cells to adapt to different energy sources available in individual organs. Each host organ poses various challenges to colonizing tumour cells in terms of availability of energy sources and oxygen or oxidative stress [10,194]. This means that those embolised cells will gain an advantage to colonise that are able to overcome metabolic barriers by metabolic plasticity, which enables them to use all resources available in an individual organ [10,197,198,199,200,201].

Thus, several biological processes are known that can trigger an SSM by seed and soil properties. Therefore, in metastatic disease, the clinical behaviour is the end product of tumour and host biology [158], both of which are not immutable constants: (1) The evolution of tumour cells can always lead to the generation of new cell clones with different biologically properties [202], which increases the chance that one of these clones will exactly match to a target organ, i.e., it is capable of metastasizing there. (2) The innate properties of the environment can be manipulated by the influence of tumour cells to form a “fertile soil”, as pmN research has shown. Whether and which of the abovementioned mechanisms is relevant for the occurrence of isPMRCC is not (yet) known at the moment.

## 10. Conclusions

The occurrence of PM in metastatic RCC—both isPMRCC and PM in multiple organ site mRCC—is coupled to clinical features that differ in some aspects from those common in solid tumour metastases, e.g., the possibility of isolated occurrence (isPMRCC), ineffectiveness of volume and growth-rate related risk factors, and better prognosis despite a larger number of affected organs. In the case of isPMRCC in particular, three special features can be detected that differ from the behaviour generally observed in extrapancreatic metastases of mRCC: (1) Genetic alterations associated with lower tumour aggressiveness; (2) The lack of influence of tumour volume and growth rate dependent prognostic factors; and (3) Immunotherapy, which is very effective in mRCC, remains ineffective in isPMRCC. Of course, it is conceivable that these three properties are completely independent of each other. However, this leads to the unsatisfactory explanation that these embolised RCC cells are equipped with an accumulation of unusual biological properties. The data presented, however, can just as well be explained by the coincidence of special RCC cells with a postulated highly specific SSM in isPMRCC. At the beginning of the process there is undoubtedly the evolution of tumour cells that leads to a multiplicity of different cell species with different cell genomes (according to the cancer evolutionary model, this cell diversity is the result of a phase of genome chaos [2,6,8,203] with following genome re-organisation [4,5,203], which is responsible not only for a diversity of cell genomes, but also for possible chromosomal rearrangements that are associated with drug resistance [6,204], drawing attention to the concept of adaptative therapy [4,6,205]). It is a specificity of the isPMRCC entity that those few circulating tumour cells that eventually settle and develop into manifest metastases are linked with reduced aggressiveness, as evidenced by genetic studies as well as the clinically proven slow progression of the disease. However, the reduced aggressiveness by itself cannot explain the phenomenon of isolated occurrence of PM and the lack of influence of risk factors. According to current knowledge, an organotropism in metastasis development—and one such is undoubtedly the isolated occurrence of PM in mRCC—is linked to an SSM. Therefore, the exclusive occurrence of metastases in one organ (pancreas) presupposes that these cells, during their evolution, are endowed not only with a low aggressiveness but also with additional properties that can trigger a highly selective SSM in host organs. This favours metastasis settlement in individual organs and prevents it in others. Therefore, in isPMRCC too, the clinical behaviour is the common end product of tumour and host biology [159].

Which biochemical alterations of the tumour cells and/or of the host organs trigger such an SSM in isPMRCC is currently unknown and reserved for future investigations. Though isolated PM of the RCC are very rare, they deserve increased investigations as there are indications that clinical course is determined—in addition to genetic changes— to an unusually high degree by an SSM too. An elucidation of the biochemical changes that control this SSM could lead to a deeper understanding of the processes that promote or prevent the colonisation of metastatic tumour cells.

## Figures and Tables

**Table 1:** Side of the primary renal cell carcinoma (RCC) and the location of metastases within the pancreas (*n* = 182; *p* = 0.707); (isPMRCC = isolated pancreatic metastases of renal cell carcinoma.)

Localisation of the isPMRCC	Side of RCC		
	Left	Right	Bilateral
Caput	51	44	2
Corpus	20	23	1
Cauda	25	15	1
Total	96	82	4

**Table 2:** isPMRCC—Impact of potential risk factors for overall survival (OS) after pancreatic surgery; (*n* = number of patients; Sing/Mult = singular vs. multiple pancreatic metastases (PM), Syn/Metachr = synchronous vs. metachronous; DFI = disease-free interval between RCC and PM surgery; Peripancr. LN = peripancreatic lymph nodes; n.s. = non-significant; *p* = *p*-value).

Authors	Year	*n*	Sing/Mult	Size	Syn/Metachr	DFI	Peripancr. LN
Institutions reports							
Milanetto [57]	2020	36	n.s.; *p* 0.61	n.s.; *p* 0.30	*p* 0.01	n.s.; *p* 0.96	*p* 0.005
Di Franco [56]	2020	21	n.s.; *p* 0.391	n.s.; *p* 0.569		n.s.; *p* 0.143	n.s.; *p* 0.07
Anderson [47]	2019	29	n.s.				
Benhaim [30]	2015	20	n.s.				
Schwarz [68]	2014	62	n.s.; *p* 0.9			n.s.; *p* 0.73	*p* 0.009
Tosoian [69]	2014	42	n.s.; *p* 0.727	n.s.; *p* 0.602	n.s.; *p* 0.509	n.s.; *p* 0.738	n.s.; *p* 0.085
Konstantinidis [35]	2010	20	n.s.; *p* 0.87	n.s.; *p* 0.87			
Reddy [38]	2008	21		n.s.; *p* 0.13	n.s.; *p* 0.98		*p* 0.01
Literature compilations							
Sellner [51]	2020	527	n.s.; *p* 0.350	n.s.; *p* 0.423	n.s.; *p* 0.790	n.s.; *p* 0.786	
Dong [65]	2016	193	n.s.; *p* 0.67	n.s.; *p* 0.87	n.s.; *p* 0.91	n.s.; *p* 0.53	*p* 0.03
Masetti [111]	2010	157	n.s.; *p* 0.862		n.s.; *p* 0.092	*p* 0.017	
Tanis [72]	2009	311	n.s.; *p* 0.892	n.s.; *p* 0.894	n.s.; *p* 0.528	n.s.; *p* 0.528	n.s.; *p* 0.85

**Table 3:** Site metastatic RCC with and without PM and survival; (*N* = size of the PM group; *n* = number of metastatic organ sites in the PM group and in control (non-PM) group).

Author, Year	*N*	Number (*n*) of Affected Organs and Frequency (%)			Median Survival (Months) Control vs. PM Group
		*n*	Control Group	PM Group	Significance
Singla [58], 2020	31				35 vs. 101
					*p* < 0.001
Chrom [139], 2018	34	1–2	52%	38%	23 vs. 46
		3, >3	48%	62%	*p* = 0.022
Kalra [142], 2016	44	1	31%	9%	26 vs. 39
		2	40%	23%	*p* < 0.01
		3, >3	29%	68%	
Yuasa [67], 2015	20				*p* < 0.0001
Grassi [22], 2013	24	1	37%	25%	23 vs. 39
		2	35%	21%	*p* = 0.0004
		3, >3	28%	54%	

**Table 4:** Mechanism leading to organotropism in metastases settlement.

Pre-metastatic niche
Organ-specific differences in immunosurveillance
Chemokine receptor mechanism
Metabolic adaptation of tumour cells

## Data Availability

The literature search was based on the PubMed^®^ registry.

## Supplementary Materials

The following are available online at https://www.mdpi.com/2072-6694/13/6/1342/s1, Table S1: Interval between RCC surgery and occurrence of pancreatic metastases, Table S2: Frequency of multiple isPMRCC, Table S3: 5-year survival rates and median survival times following surgical treatment of isPMRCC, Table S4: Distant organ metastases occurring after isPMRCC surgery, Table S5: Frequency of regional LN metastases, Table S6: Liver metastases occurring after isPMRCC surgery, Table S7: Distant organ metastases occurring before isPMRCC surgery.
