# Supplementary material for: Tumour Evolution and Seed and Soil Mechanism in Pancreatic Metastases of Renal Cell Carcinoma
Source: Cancers (Basel). 2021 Mar 16;13(6):1342. doi: 10.3390/cancers13061342 (PMC8002056; doi:10.3390/cancers13061342)
Supplement: Supplementary file 1 [file cancers-13-01342-s001.pdf]

cancers MDPI

Supplementary Material: Tumour Evolution and Seed and Soil Mechanism in Pancreatic Metastases of Renal Cell Carcinoma

Franz Sellner, Sabine Thalhammer and Martin Klimpfinger

Table S1. Interval between RCC surgery and occurrence of pancreatic metastases (metachronous cases).

Author	Year	Interval (y)
Milanetto [57]	2020	7
Fahlbusch [27]	2020	14
Chikhladze [28]	2020	10
Di Franco [56]	2020	6.9
Anderson [47]	2020	8
Ma [63]	2019	11
Chatzizacharias [64]	2017	6.7
Dong [65]	2016	6,9
Fikatas [66]	2016	10.2
Yuasa [67]	2015	7.8
Benhaim [30]	2015	10.8
Kimura [32]	2014	8.4
Moletta [33]	2014	8.2
Schwarz [68]	2014	9,8
Tosoian [69]	2014	11.2
Niess [70]	2013	8.2
Alzahrani [44]	2012	7.8
Yazbek [71]	2012	11.4
You [34]	2011	5.3
Konstantinidis [35]	2010	8.7
Volk [83]	2009	7.8
Tanis [72]	2009	9.8
Schauer [39]	2008	9.2
Akatsu [40]	2007	7.0
Eidt [73]	2007	13
Crippa [74]	2006	5.5
Wente [41]	2005	7.2
Bassi [75]	2003	10.5
Law [76]	2003	6.9
Peschaud [77]	2002	8.7
Thompson [78]	2000	8.4
Ghavamian [79]	2000	9.7
Kassabian [80]	2000	12

Table S2. Frequency of multiple isPMRCC (*N* = number of reported isPMRCC cases; % = frequency of multiple PM).

Author	Year	N	%
Bauschke [62]	2021	16	19
Milanetto [57]	2020	39	54
Anderson [47]	2020	29	48
Di Franco [56]	2020	21	62
Chikhladze [28]	2020	20	40
Ma [63]	2019	13	31
Benhaim [30]	2015	20	35
Moletta [33]	2014	8	50
Schwarz [68]	2014	62	37
Yazbek [71]	2012	11	9
You [34]	2011	7	28
Konstandinidis [35]	2010	20	25
Tanis [72]	2009	10	30
Volk [83]	2009	14	28
Schauer [39]	2008	10	70
Zerbi [84]	2008	23	28
Wente [41]	2005	15	33
Bassi [75]	2003	17	47
Law [76]	2003	14	36
Peschaud [77]	2002	7	29
Ghavamian [79]	2000	23	48
Kassabian [80]	2000	5	60
Thompson [78]	2000	21	43

Table S3. 5-year survival rates (5y SVR) and median survival times (ST) following surgical treatment of isPMRCC.

Author, Year	N	Follow up Time (months)	5y SVR (%)	ST (years)
Bauschke [62], 2021	16		68	5.4
Milanetto [57], 2020	39	68	79**	
Fahlbusch [27], 2020	12		84*	
Di Franco [56], 2020	21	77	72	6.3
Chikhladze [28], 2020	20	76	89	
Anderson [47], 2020	29	64	80*	
Ito [46], 2018	58		70**	
Madkhali [43], 2018	17		50	
Chatzizacharias [64], 2017	7	33	71	7.8
Fikatas [66], 2016	19	49	71	
Yuasa [67], 2015	20	42	79	
Benhaim [30], 2015	20	69	72*	
Wiltberger [31], 2015	10	76	60	
Untch [24], 2014	27	30		8.0
Tosoian [69], 2014	42	84	52	5.5
Schwarz [68], 2014	62	91	63**	4.4
Kimura [32], 2014	13	67	77	
Yazbek [71], 2012	11			6.5
Konstandinidis [35], 2010	20	38	61	8.7
You [34], 2010	7		66	8.75
Volk [83], 2009	14			6.2
Zerbi [84], 2008	23	31	88	3.7
Schauer [39], 2008	10	56		6.2
Bahra [85], 2008	9		100	
Eidt [73], 2007	7			4.3
Crippa [74], 2006	5		80	3.4
Wente [41], 2005	12		53	
Law [76], 2003	14	32	75**	
Bassi [75], 2003	22	33	53	
Sohn [101], 2001	10		75	
Faure [42], 2001				4.0
Ghavamian [79], 2000	11			4.6
Thompson [78], 2000	21		43	6.2

* 4-year survival rate; ** multicentre report.

Table S4. Distant organ metastases occurring after isPMRCC surgery (*N* = number of observations);

Author	Year	*N*	Site
Chikhladze [28]	2020	7	Contralat. kidney [1]; muscle; bone/adrenal/lung; lung/bone; liver/lung; spinal cord/kidney [1]; LN [1]
Schammel [55]	2020	1	Pancreatic remnant [1]
Glinka [53]	2019	4	Lung; bone; brain/bone; bone
Yagi [23]	2017	2	Lung; lung
Shatveryan [116]	2017	1	Lung
Nihei [115]	2016	1	Pancreas caput [1]
Chang [114]	2015	1	Pancreatic remnant [1]
Takeshi [113]	2014	1	Cauda panc. [1]
Moletta [33]	2014	5	Liver, liver, thyroid, lung, pancreas [1]
Macri [52]	2014	1	Pancreas corpus, [1]
Lauro [112]	2014	1	Lung
Espinoza [100]	2014	2	Lung, lung
Yoshikawa [98]	2013	1	Pancreatic remnant [1]
Niess [70]	2013	10	Lung/bone/liver; brain/lung; lung/parotid; lung/liver; thyroid/cerebellum; mediastinum/heart/brain; liver; lung/brain; liver/bone; lung
Hata [97]	2013	1	Duodenum
Yazbek [71]	2012	5	Local [1]; pancreas [1]; distant pancreas [1]; distant pancreas [1]; diffuse; distant pancreas [1]
You [34]	2011	5	Brain; lung; liver/lung; adrenal[1]/lung; lung
Watanabe [94]	2011	2	Liver; liver/lung
Masetti [111]	2010	1	Lung
Machado [110]	2009	1	Bone
Deguchi [93]	2009	7	Thyroid; lung/LN [1]; lung/bone/liver; lung; contralat. kidney [1]/lung; liver/GI tract; pituitary/bone
Zerbi [84]	2008	11	Lung; lung; lung; lung; liver; liver; retroperit. LN [1]; brain; lung/brain; psoas; pancreas [1]
Schauer [39]	2008	3	Diffuse
Karimi [109]	2007	2	Diffuse
Eidt [73]	2007	1	Diffuse
Köhler [92]	2006	1	Liver
Sellner [21]	2006	5	Pancreas[1]; pancreas[1]; skin; thyroid; liver/adrenal
Ninan [108]	2005	1	Thyroid
Minni [88]	2004	1	Adrenal [1]
Zacharoulis [107]	2003	2	Bone; lung
Law [76]	2003	8	Pancreas [1]; lung; retroperit.LN[1]; pancreas [1]; distant pancreas [1]; pancreas [1]; lung; pancreas [1]
Bechade [99]	2002	2	Thyroid, lung
Sohn [101]	2001	1	Soft tissue
Faure [42]	2001	3	Liver/lung/brain; liver/lung/brain; bone
Le Borgne [91]	2000	1	Liver
Kassabian [80]	2000	2	Lung; pancreas/liver;
Ghavamian [79]	2000	6	Mesentery [1]; lung; chest wall; lung; adrenal [1]; omentum [1]
Yavaşçaoğlu [106]	1999	1	Periprostatic
Merkle [90]	1998	1	Liver
Hashimoto [86]	1998	3	Multiple; skin; mediast. LN[1]/lung
Butturini [89]	1998	1	Liver
Altschuler [96]	1998	1	Lung
Onishi [105]	1995	1	Multiple
Fabre [104]	1995	1	Brain

Marcote [103]	1993	1	Bone
Oka [102]	1991	1	Pituiary
Simpson [95]	1989	1	Thyroid
Py [87]	1984	2	Bone/liver; thyroid/liver/pancreas
Total		124	haematogenous metastases 93 75%<br>pancreatic metastases 19 15.3%

[1] Metastases that are not unequivocally attributable to a systemic haematogenous spread.

Table S5. Frequency (%) of regional LN metastases; *n* = cases with LN, *N* = operated isPMRCC;

Year	Author	*n*	N	%
Institution reports				
2020	Milanetto [57]	5	39	12.8
2020	Di Franco [56]	5	21	23.8
2020	Chikhladze [28]	0	11	0
2018	Madkhali [43]	0	17	0
2015	Benhaim [30]	0	20	0
2014	Kimura [32]	0	13	0
2014	Tosoian [69]	2	39	5.1
2014	Schwarz [68]	9	33	27.3
2010	Konstantinidis [35]	3	12	25
2008	Zerbi [84]	0	23	0
2007	Eidt [73]	0	7	0
2005	Wente [41]	0	15	0
2004	Moussa [122]	0	22	0
2003	Bassi [75]	0	22	0
Casuistic reports			187	
2015	Gajendra [123]	1		
2012	Yazbek [71]	3		
2004	Minni [88]	1		
2003	Law [76]	1		
		30	481	6.2

Table S6. Liver metastases occurring after isPMRCC surgery;

Author	Year	*N*
Chikhladze [28]	2020	1
Brozetti [124]	2019	1
Moletta [33]	2014	2
Niess [70]	2013	4
You [34]	2011	1
Watanabe [94]	2011	2
Deguchi [93]	2009	2
Zerbi [84]	2008	2
Köhler [92]	2006	1
Sellner [21]	2006	1
Faure [42]	2001	2
Le Borgne [91]	2000	1
Kassabian [80]	2000	1
Merkle [90]	1998	1
Butturini [89]	1998	1
Py [87]	1984	2
Total		25

Table S7. Distant organ metastases occurring before isPMRCC surgery.

Author	Year	*N*	Localisation
Chikhladze [28]	2020	4	Contralat. kidney[1], thyroid, lung, paratid
Wakabayashi [133]	2019	1	Lung
Yamashita [132]	2018	1	Adrenal[1]/thyroid
Yagi [23]	2017	1	Lung
Boussios [131]	2016	1	Hepar
Kitade [130]	2015	1	Chest wall/thyroid
Yoshikawa [98]	2013	1	Contralat. kidney[1]
Niess [70]	2013	4	Lung/peritoneum, lung/thyroid, cerebellum; lung
Hata [97]	2013	1	Thoracic vertebrae
Zygulska [129]	2012	1	Gallbladder
Yazbek [71]	2012	2	Lung, adrenal[1]
You [34]	2011	1	Thyroid
Mourra [36]	2010	2	brain; lung
Deguchi [93]	2009	4	Lung/LN[1]; lung; lung; lung/pituitary,
Zerbi [84]	2008	4	Lung; brain; thyroid; muscle
Schauer [39]	2008	4	Adrenal/local[1]; thyroid/breast; local[1]; thyroid
Maeda [128]	2007	1	Lung
Bassi [75]	2003	4	Thyroid; thyroid; adrenal[1]; contralat. kidney[1]
Mehta [125]	2000	1	Lung
Hashimoto [86]	1998	1	Bowel
Altschuler [96]	1998	1	Lung
Adem [127]	1998	1	Hepar/cerebellum
Barras [126]	1996	1	Lung
Simpson [95]	1989	1	Contralat. kidney[1]
Strijk [118]	1989	1	Skin
Total		45	Hematogenous metastases 35 (78%]

[1] Metastases that can be attributed to a local metastatic pathway.
